# Supplementary material for: Dynamic transcriptomic profiles of zebrafish gills in response to zinc supplementation
Source: BMC Genomics. 2010 Oct 11;11:553. doi: 10.1186/1471-2164-11-553 (PMC3091702; doi:10.1186/1471-2164-11-553)
Supplement: Additional file 2 — Interactive Direct Interaction Network representing the molecular interactions between zinc, copper, iron, calcium and proteins encoded by transcripts changed by zinc supplementation. Mini web-site containing index.html and hyperlinked pages in subdirectory describing a Direct Interaction Network automatically generated based on curated interactions contained within the proprietary PathwayArchitect database. Ovals represent proteins and the circles symbolize metal ions. Objects are coloured by their abundance in zebrafish at the time-point they were significantly different from the control is a scale from -4 fold (dark green) to +4 fold (dark red). Where significant differences were found at more than one time-point, the colour overlay shows expression at the first instance. Dark blue squares denote 'binding', and light blue squares 'expression'; green squares stand for 'regulation', green diamonds for 'metabolism', and green circles for 'promoter binding'. Arrow heads indicate directionality of the interaction where annotated. All nodes and edges can be further interrogated by selecting the relative area of the image. [file 1471-2164-11-553-S2.zip › PathwayArchitect Zn xs DIN/470999.html]

# REGULATION:

|  |  |
| --- | --- |
| Type | REGULATION |
| Effect | Positive |


---

|  |  |
| --- | --- |
| Score | 0 |


---

|  |  |
| --- | --- |
| Reference Count | 10 |


---

|  |  |
| --- | --- |
| Mechanism | Unknown |


---

|  |  |
| --- | --- |
| Reference:0 || Sentence | "Unlike zinc, copper in the serum and that associated with metallothionein showed little response to endotoxin." |
| PMID | 6388345 |
| Year | 1984 |
| Species | Rat |
| Journal | Am J Physiol |
| RefScore | 1 |
| Source | PArchNLP |
  |
|  |  |


---

|  |  |
| --- | --- |
 Reference:1 || Sentence | "All the intestines incubated in the low copper media, regardless of rat zinc status, had the majority of copper associated with MT." |
| PMID | 6822916 |
| Year | 1983 |
| Species | Rat |
| Journal | J Nutr |
| RefScore | 2 |
| Source | PArchNLP |
  ||  |  |


---

|  |  |
| --- | --- |
 Reference:2 || Sentence | "We have examined the relationship between (a) histochemically demonstrable copper using rubeanic acid, (b) copper-associated protein (CAP) using orcein, and (c) immunoreactive metallothionein (MT) using DNP hapten sandwich staining and have correlated these with histological lesions in 95 liver biopsies from patients with conditions associated with hepatic copper retention, 4 fetal livers, and 25 histologically normal adult controls." |
| PMID | 2754543 |
| Year | 1989 |
| Species | Human |
| Journal | J Pathol |
| RefScore | 1 |
| Source | PArchNLP |
  ||  |  |


---

|  |  |
| --- | --- |
 Reference:3 || Sentence | "Experiments with primary cultures enriched in neurons or astrocytes indicate that MT is present in both cell types and that responds to the well-known MT inducers zinc, copper and glucocorticoids." |
| PMID | 7834015 |
| Year | 1994 |
| Species | Human |
|  | Rat |
| Journal | Biol Signals |
| RefScore | 1 |
| Source | PArchNLP |
  ||  |  |


---

|  |  |
| --- | --- |
 Reference:4 || Sentence | "The cadmium exposure increased hepatic and renal zinc and renal copper levels, probably as a result of cadmium-induced MT, and some of the levels were normalized considerably by the subsequent treatment with cysteine, DMPS or to a lesser extent N-acetyl cysteine and their combinations, showing their protective effects against cadmium toxicity." |
| PMID | 11807931 |
| Year | 2002 |
| Species | Rat |
| Journal | J Appl Toxicol |
| RefScore | 0 |
| Source | PArchNLP |
  ||  |  |


---

|  |  |
| --- | --- |
 Reference:5 || Sentence | "In JIB rats, however, liver copper, MT protein, and MT mRNA were significantly elevated, and a high proportion of the intracellular zinc and copper was associated with MT." |
| PMID | 11090006 |
| Year | 2000 |
| Species | Rat |
| Journal | Am Surg |
| RefScore | 2 |
| Source | PArchNLP |
  ||  |  |


---

|  |  |
| --- | --- |
 Reference:6 || Sentence | "Compared to controls, liver and kidneys of diabetic dams showed an increased concentration of zinc and copper that was associated with metallothionein." |
| PMID | 2484570 |
| Year | 1988 |
| Species | Rat |
| Journal | Biol Trace Elem Res |
| RefScore | 2 |
| Source | PArchNLP |
  ||  |  |


---

|  |  |
| --- | --- |
 Reference:7 || Sentence | "The contents of zinc, copper and cadmium, which are primary inducers of MT, in the incubation medium of macrophages in the presence of endotoxin were not different from those in the absence of endotoxin." |
| PMID | 3494383 |
| Year | 1987 |
| Species | Rat |
| Journal | Acta Med Okayama |
| RefScore | 0 |
| Source | PArchNLP |
  ||  |  |


---

|  |  |
| --- | --- |
 Reference:8 || Sentence | "Injection of zinc and also of cadmium and copper had the opposite effect and increased MT-I concentrations in both samples, although the effects of zinc on blood cells and urine were relatively transient." |
| PMID | 3309208 |
| Year | 1987 |
| Species | Rat |
| Journal | J Nutr |
| RefScore | 2 |
| Source | PArchNLP |
  ||  |  |


---

|  |  |
| --- | --- |
 Reference:9 || Sentence | "The levels of copper-induced MT mRNAs remained at a plateau for up to 15 h." |
| PMID | 2555705 |
| Year | 1989 |
| Species | Human |
| Journal | Mol Cell Biol |
| RefScore | 1 |
| Source | PArchNLP |
  |


---

|  |  |
| --- | --- |
